# SVM-Based Model Combining Patients’ Reported Outcomes and Lymphocyte Phenotypes of Depression in Systemic Lupus Erythematosus

**DOI:** 10.3390/biom13050723

**Published:** 2023-04-23

**Authors:** Chen Dong, Nengjie Yang, Rui Zhao, Ying Yang, Xixi Gu, Ting Fu, Chi Sun, Zhifeng Gu

**Affiliations:** 1 Department of Rheumatology, Affiliated Hospital of Nantong University, Medical School of Nantong University, Nantong University, Nantong 226001, China; 2 Department of Geriatrics, Affiliated Hospital of Nantong University, Nantong University, Nantong 226001, China

**Keywords:** systemic lupus erythematosus, depression, lymphocyte phenotype, patients’ reported outcomes, SVM model, logistic regression model

## Abstract

Background: The incidence of depression in patients with systemic lupus erythematosus (SLE) is high and leads to a lower quality of life than that in undepressed SLE patients and healthy individuals. The causes of SLE depression are still unclear. Methods: A total of 94 SLE patients were involved in this study. A series of questionnaires (Hospital Depression Scale, Social Support Rate Scale and so on) were applied. Flow cytometry was used to test the different stages and types of T cells and B cells in peripheral blood mononuclear cells. Univariate and multivariate analyses were conducted to explore the key contributors to depression in SLE. Support Vector Machine (SVM) learning was applied to form the prediction model. Results: Depressed SLE patients showed lower objective support, severer fatigue, worse sleep quality and higher percentages of ASC%PBMC, ASC%CD19+, MAIT, TEM%Th, TEMRA%Th, CD45RA+CD27-Th, TEMRA%CD8 than non-depressed patients. A learning-based SVM model combining objective and patient-reported variables showed that fatigue, objective support, ASC%CD19+, TEM%Th and TEMRA%CD8 were the main contributing factors to depression in SLE. With the SVM model, the weight of TEM%Th was 0.17, which is the highest among objective variables, and the weight of fatigue was 0.137, which was the highest among variables of patients’ reported outcomes. Conclusions: Both patient-reported factors and immunological factors could be involved in the occurrence and development of depression in SLE. Scientists can explore the mechanism of depression in SLE or other psychological diseases from the above perspective.

## 1. Introduction

Systemic lupus erythematosus (SLE) is a chronic inflammatory autoimmune disease characterized by the abnormal activation of T and B lymphocytes with decreased quality of life [1,2]. SLE affects multiple organs and systems and is especially associated with neuropsychiatric problems. Depression is the most common neuropsychological symptom in SLE patients, and the incidence is 8.7–78.6% [3]. Depression is associated with fatigue, sleep disorders, sexual function disorders, reduced adherence, and even suicide ideation and behavior, which seriously affect the prognosis of SLE patients [4,5,6,7,8,9]. Despite the high incidence and poor prognosis of depression, there are no effective treatment strategies or satisfactory early identification methods.

An early diagnosis and intervention of depression are advantageous for controlling disease activity, reducing medical costs and improving the prognosis. Thus, the “risk prediction” strategy will be more helpful to manage depression in the early stage than the traditional “treatment needs assessment”, which is conducive to timely interventions to reduce other impairments caused by depression. Additionally, exposure risks in the early stage are important factors for the risk prediction of depression. The occurrence and development of depression is a multi-step, time-dependent process caused by many factors, and no single factor can explain the outcome of depression in SLE patients. Moreover, it was reported that the predictive performance of a single factor was worse than that of multivariate predictive models [10,11]. Therefore, a depression risk prediction model should be constructed in conjunction with multidimensional factors to improve its sensitivity and specificity. In recent years, many socioeconomic factors and other patient-reported indicators (such as educational level, income, fatigue, disease duration and so on) have been observed to be significantly correlated with SLE depression [12,13], which will provide helpful information for model construction. However, there are still some limitations, such as a lack of objective parameters and optimized modeling methods.

The roles of lymphocyte abnormalities in neuropsychiatric diseases have been widely explored, especially in depression. The abnormal expression of T cells participated in the development of depression [14]. CD3+CD8+, CD4+ and CD3+CD25+ T-cell subsets were significantly unbalanced in patients with major depression, especially in women [15,16]. The imbalance of Th1/Th2 cells can be alleviated by interventions for depression in SLE patients [17]. At the level of animal models, depressive behavior is spontaneous in MRL/lpr transgenic mice, which is the most common SLE animal model and characterized by the abnormal activation of T and B cells. In addition, pristane-induced lupus mice, which is another useful model for SLE, exhibit a series of neuropsychiatric behavioral deficits and pathological changes in the brain [18]. Furthermore, it was found that the stress-induced metabolic disorder of CD4+ T cells can lead to anxiety-like behavior [19]. These studies indicated the key role of lymphocytes in neuropsychiatric diseases. At present, few studies have focused on the association between lymphocytes and depression, let alone the combination of lymphocytes with multidimensional factors. This project intended to explore the contributing factors and models of SLE depression from the above perspectives, using both objective lymphocyte indicators and patients’ subjective reports.

## 2. Materials and Methods

### 2.1. Participants

SLE patients who met the 1997 ACR and 2012 SLICC classification criteria were invited to participate in this study at the Affiliated Hospital of Nantong University from October 2018 to November 2019. Individuals were excluded if they had any of the following characteristics: (1) less than 18 years old; (2) inability to cooperate or failure to complete questionnaires; (3) serious cognitive impairment, epilepsy or stroke or taking central nervous system drugs; (4) major personal or family events in last 3 months, such as divorce, bereavement, etc., (5) complications with other rheumatic diseases, such as rheumatoid arthritis, fibromyalgia and so on. The study was approved by the Ethics Committee of the Affiliated Hospital of Nantong University (2017-K003), and written informed consent was obtained from all the participants, in accordance with the Declaration of Helsinki.

### 2.2. Demographics and Clinical Characteristics

Socioeconomic variables (age, gender, BMI, place of residence, marital status, educational level, employment, income, etc.) were assessed using a self-designed questionnaire.

### 2.3. Patients’ Reported Outcomes

The Hospital Anxiety and Depression Scale (HADS), Pittsburgh Sleep Quality Index (PSQI), Multidimensional Fatigue Inventory (MFI-20), Social Support Rate Scale (SSRS) and Simplified Coping Style Questionnaire (SCSQ) were used to assess depression, sleep quality, fatigue, social support and coping style. The results were added to a computer database by two research assistants and were double-checked against the original data prior to analysis.

### 2.4. Objective Evaluation Variables

Clinical variables (disease course and disease activity (SLEDAI score)) and antibody and biochemical indicators (ESR, CRP, C3, C4, anti-dsDNA and others) were obtained from patients and medical records. T- and B-lymphocyte subsets were obtained as follows: Morning blood samples were obtained from the participants, and peripheral blood mononuclear cells (PBMCs) were isolated by density centrifugation using Ficoll-Paque™ PLUS. After centrifugation, the mononuclear cell layer containing PBMCs was removed and added to a new universal tube, and the cells were washed twice with MACS buffer. For the phenotypic characterization of T cells and B cells, isolated PBMCs were stained with a combination of fluorochrome-conjugated antibodies. Our panel enabled the survey phenotyping of T-cell subsets, as well as B cells. Up to 1 × 10^6^ PBMCs were washed twice with cold MACS at 500 g for 10 min at 4 °C. Surface staining reactions were performed using light-protected incubation with 50 µL of surface antibody cocktails for 15 min at room temperature. After washing twice with MACS buffer at 500 g for 10 min at 4 °C, surface-stained cells were resuspended in 200 µL of fixation buffer, incubated for 20 min at room temperature, washed twice with MACS buffer at 500 g for 10 min at 4 °C and resuspended in 250 µL of FACS buffer for acquisition. Data were acquired using a BD FACS Fortessa flow cytometer. Data analysis and plotting were performed using FlowJo v9.

### 2.5. Data Analysis

For continuous data, means and standard deviations (SDs) or medians (IQR) were calculated, and group differences (depression versus non-depression according to the HADS) were analyzed by an independent sample *t*-test or the Mann–Whitney test. Descriptive statistics also involved frequencies (%) for categorical variables, and group differences were assessed using the chi-square test. All variables with significant differences in the depressed condition (*p*-value < 0.05, two-sided) in univariate tests served as alternative features for the depression risk prediction model. The above analyses were performed using SPSS version 21.0.

Support Vector Machine (SVM) is one of the many machine learning methods used for classification, regression and outlier detection and is a powerful method for recognizing subtle patterns in complex datasets. In recent years, the SVM technique has been widely used in medicine and ophthalmology [20,21]. In this study, we developed a multidimensional SLE depression risk prediction model using SVM. The main features used for training and testing were selected by performing significance tests on the variables. The SVM analysis to construct the SLE depression prediction model was performed using the R package “e1071”. The receiver operator characteristic (ROC) curve and area under the curve (AUC) were used to measure the performance of the models.

## 3. Results

In this study, the clinical data of 94 SLE patients with an average age of 39.09 ± 13.70 and a median disease duration of 5 years were analyzed. We divided all SLE patients into depression and non-depression groups according to their HADS-D scores, and 19.1% of SLE patients were depressed. Depressed SLE patients were characterized by more fatigue and poor sleep quality. Lower objective support in depressed SLE patients was also observed, and men with lupus had a higher rate of depression. However, there were no significant differences in educational level, income, coping style or other socioeconomic or patient-reported factors of SLE among the 94 SLE patients (Table 1).

In addition, we analyzed more objective data of these patients, such as biochemical and antibody indexes. After analyzing routine blood parameters, liver function, kidney function, SLEDAI, some inflammation indexes and C3 and C4 complement, we found no statistical differences in any of these variables, which is shown in Table 2.

In order to explore the role of immune cells in SLE depression, we estimated and analyzed a total of 96 types or stages of T and B lymphocytes. Considering the individual differences in cell abundance and the limited sample size of this study, we used an abnormal distribution test to analyze all T- and B-lymphocyte subsets. Using the Mann–Whitney test, we found that ASC%PBMC and ASC%CD19+ were significantly enriched in depressed SLE patients, and the distribution of the other 20 B-lymphocyte subjects showed no significant differences. The above findings are shown in Table 3.

In terms of T cells, the abundance of MAIT, TEM%Th, TEMRA%Th, CD45RA+CD27-Th and TEMRA%CD8 in depressed SLE patients was also higher than in non-depressed patients, and no significant differences were observed in the other CD4, CD8, Th and Treg subsets. The comparison results of T lymphocytes between depressed and non-depressed SLE patients are presented in Table 4.

Accurately estimating the incidence of SLE depression is important because it plays a crucial role in the treatment approach, medical resource allocation and effective communication with patients. We found that SVM generally outperformed linear regression when applied to SLE depression prediction. These results will increase the effectiveness of SLE treatment. We analyzed the SVM model with R packages, and the weight of each variable was evaluated. Finally, we found that the weights of fatigue, objective support, ASC%CD19+, TEM%Th and TEMRA%CD8 were higher than 0.1. Among these variables, TEM%Th was the most important variable, with a weight of 0.170. The detailed weights of these 11 indicators are shown in Table 5 and Table 6.

**Figure 1:**
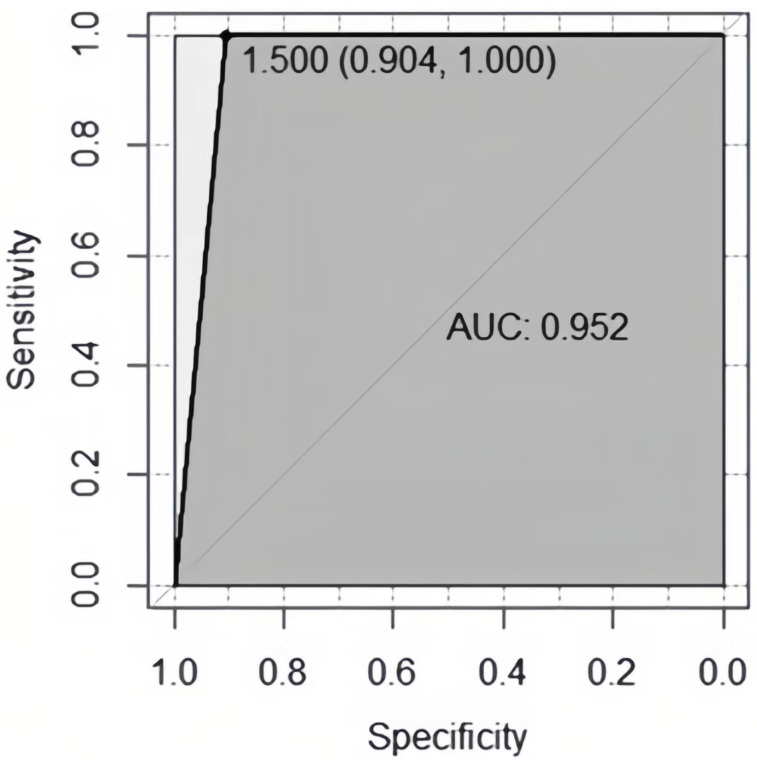
The ROC curve of SVM-based SLE depression prediction model.

The ROC curve and AUC were used to estimate the performance of the models. The AUC of the SVM model was 0.952, which indicated that the SLE depression model based on SVM had higher prediction accuracy (Figure 1).

## 4. Discussion

The risk prediction of SLE depression may be important in preventing its deleterious short-term and long-term consequences. In this study, the incidence rate of depression in SLE was 19.1%. Furthermore, depressed SLE patients were more prone to fatigue and poor sleep quality. Increasing evidence indicates that fatigue is closely associated with depression in SLE patients and is even a key mediating variable [22]. However, fatigue is also a common symptom in SLE patients, and as many as 95% of SLE patients have been found to suffer from fatigue [23]. It has also been reported that sleep disorders are common among SLE patients and are not associated with depression [24]. Therefore, as an autoimmune disease, the prediction of depression in SLE patients should not be based solely on the patient’s self-reported outcomes but also include the patient’s objective indicators, such as biochemical and inflammatory markers, immune status and others.

In this study, we did not find statistical significance between depression and blood biochemical indexes. In terms of immune indicators, based on the notion that T cells may play a role in neuroprotection and the inflammatory reaction process during stress and inflammation, impaired T-cell function may directly contribute to the development of depression [25,26]. To our knowledge, there is only one recent study that used multiparameter flow cytometry to compare circulating lymphocyte subsets in SLE patients with or without depression. Multiparameter flow cytometry immunophenotyping has been a mainstay in the diagnosis and monitoring of hematologic cytological changes. Multiparameter flow cytometry immunophenotyping is well-known to provide an accurate assessment of the expression of multiple markers and their fluorescence intensity in thousands of individual cells. Its convenience is shown in the clear discrimination between aberrant and both normal and reactive cells, even when they are present at low or very low frequencies in a sample [27]. A study used multiparameter flow cytometry to identify CD4+CD25+ Tregs and CD4+ CD25+FOXP3+ Tregs in major depressive disorder [28].

We found that ASC%PBMC, ASC%CD19+, MAIT, TEM%Th, TEMRA%Th, CD45RA+CD27-Th and TEMRA%CD8 in depressed SLE patients were also higher than in non-depressed patients. Several studies have focused on the role of MAIT cells in psychiatric illnesses. Accumulated evidence revealed that elevated MAIT cells were closely related to schizophrenia [29]. Although the number of MAIT cells in the brain is low compared to that in the blood, their activation could cause severe central nervous system lesions, suggesting that the small number of infiltrating MAIT cells may exert deleterious functions in situ [30]. Similarly, the abundance of MAIT cells was higher in depressed SLE patients than in the non-depressed group in the present study. Interestingly, a study demonstrated that MAIT cells were numerically deficient in SLE, but the enhanced function was associated with lupus disease activity [31,32]. It was assumed that the increase in MAIT cells was attributable to depression but not SLE, which indicates the possible key role of MAIT cells in neuropsychological diseases. TEM has been considered to be persistent and active in autoimmune diseases, but its role in depression is unclear [33]. It was reported that a high number of TEM cells could induce inflammation, demyelination and neuronal cell death in the central nervous system [34]. Peripheral CD8+TEMRA cells were increased in patients with Alzheimer’s disease and were negatively associated with neurocognitive function because of their ability to secrete proinflammatory cytokines in the peripheral immune system [35]. Although the relationship between humoral immunity and depression has not been directly demonstrated, multiple interactions with cellular immunity may be correlated with the severity of depression [36]. Our study demonstrated once again that lymphocytes were involved in the development of depression in SLE and can serve as a predictive marker of depression in SLE. In our results, the roles of some types of lymphocyte subsets in psychological impairment are still unclear. Therefore, the specific mechanism of these cells in SLE depression needs further study.

Accurately estimating the occurrence of depression in SLE patients is important because it plays a crucial role in the processes of disease treatment, prognosis and communication with patients. SVM is a useful classification method for prediction. SVM with non-linear data may afford a better performance of prediction models than other modeling methods. SVM with non-linear kernels can generate more complex, multidimensional decision boundaries, which may lead to the better performance of prediction models [37].

## 5. Conclusions

In this work, we tested the ability of an SVM model to predict depression incidence in SLE patients using demographic sociological data, patient-reported data, and biochemical and lymphocyte subsets detected by multiparameter flow cytometry. Overall, we observed the suitable predictive ability of SVM. We also applied the criticality of lymphocyte subsets in SLE depression prediction. These findings can inform new strategies for psychological status and other applications. However, in this study, the SLICC/ACR damage index for lupus-related organ damage was not included, which is a limitation of this study. The relationship between organ damage, immune subtypes and depression is worthy of in-depth exploration in the future.

## Figures and Tables

**Table 1:** Differences in patients’ reported parameters between SLE patients with and without depression.

	All SLE Patients (94 Samples)	Depression (18 Samples)	Non-Depression (76 Samples)	*p*
Age, years ^a^	39.09 ± 13.70	37.5 ± 11.18	39.46 ± 14.27	0.588
Gender, women ^b^	87 (92.6)	14 (77.8)	73 (96.1)	0.008 *
BMI ^a^	22.42 ± 3.30	23.26 ± 3.85	22.21 ± 3.15	0.228
Place of residence ^b^				0.727
Urban	40 (42.6)	7 (38.9)	33 (43.4)	
Rural	54 (57.4)	11 (61.1)	43 (56.6)	
Marital status ^b^				0.148
Married	70 (74.5)	11 (61.1)	59 (77.6)	
Other	24 (25.5)	7 (38.9)	17 (22.4)	
Education level ^b^				0.703
≤9 years	59 (62.8)	12 (66.7)	47 (61.8)	
>9 years	35 (37.2)	6 (33.3)	29 (38.2)	
Employment, yes ^b^	62 (66.0)	14 (77.8)	48 (63.2)	0.239
Yearly per capita income, yuan ^b^				0.203
<15,000	41 (44.1)	5 (27.8)	36 (48.0)	
15,000–33,000	29 (31.2)	6 (33.3)	23 (30.7)	
>33,000	23 (24.7)	7 (38.9)	16 (21.3)	
Medical insurance, yes ^b^	71 (76.3)	15 (88.2)	56 (73.7)	0.202
Social support ^a^	39.95 ± 7.07	38.12 ± 7.96	40.36 ± 6.85	0.240
Objective support	8.26 ± 2.15	7.00 ± 2.45	8.56 ± 1.97	0.005 *
Subjective support	24.82 ± 5.22	24.50 ± 5.82	24.89 ± 5.10	0.776
Utilization of support	6.92 ± 2.38	7.00 ± 1.94	6.91 ± 2.48	0.885
Coping style ^a^				
Positive attitude	23.07 ± 7.84	20.78 ± 8.98	23.64 ± 7.49	0.168
Negative attitude	10.49 ± 5.57	11.00 ± 6.58	10.36 ± 5.34	0.666
Fatigue ^a^	50.35 ± 14.01	60.88 ± 13.49	47.93 ± 13.05	0.002 *
Sleep quality ^a^	5.41 ± 3.32	7.00 ± 3.77	5.01 ± 3.10	0.022 *
Prednisone, mg/qd ^a^	11.99 ± 11.95	10.17 ± 6.71	12.61 ± 13.28	0.498
Hydroxychloroquine, g/qd ^a^	0.22 ± 0.06	0.22 ± 0.06	0.22 ± 0.07	0.660

Notes: *: *p* < 0.05. ^a^: Values are presented as the mean ± SD and were analyzed by independent *t* test. ^b^: Values are presented as the number(percent) and were analyzed by chi-square test.

**Table 2:** Comparison of laboratory indexes between depressed and non-depressed SLE patients.

	SLE Patients	Depression	Non-Depression	*p*
Disease duration, years ^a^	5 (1.1, 10)	5 (1, 10)	6 (1.2, 10)	0.853
SLEDAI ^a^	6 (4, 10)	8.5 (3.75, 12)	6 (4, 9.5)	0.187
White blood cells, 10^9^/L ^a^	4.3 (3.4, 6.4)	4.9 (4.03, 5.70)	4.3 (3.28, 6.73)	0.744
Neutrophils, 10^9^/L ^a^	2.87 (2.10, 4.26)	3.43 (2.52, 4.22)	2.83 (1.92, 4.27)	0.203
Red blood cells, 10^12^/L ^a^	4.37 (4.07, 4.66)	4.47 (4.00, 4.86)	4.36 (4.07, 4.64)	0.557
Hemoglobin, g/L ^a^	126 (115, 136)	129 (110.5, 139.5)	126 (115.75, 136)	0.764
Platelets, 10^9^/L ^b^	205.59 ± 84.69	231.12 ± 61.92	199.73 ± 88.40	0.169
Urine protein, g/24 h ^b^	0.99 ± 1.24	2.00 ± 2.53	0.79 ± 0.83	0.493
Aspartate aminotransferase, μ/L ^a^	24 (20, 31)	24 (21.5, 31.5)	24 (20, 31)	0.569
Alanine aminotransferase, μ/L ^a^	22 (15.5, 32)	21 (13.5, 33)	22.5 (17.25, 32)	0.507
Albumin, g/L ^b^	40.97 ± 5.83	41.27 ± 5.35	40.91 ± 5.97	0.821
Globulin, g/L ^b^	30.47 ± 5.69	29.62 ± 5.72	30.68 ± 5.71	0.499
Creatinine, μmol/L ^a^	52 (47, 66)	53 (43, 67.75)	52 (47, 67)	0.877
C-reactive protein, mg/L ^a^	2.04 (1.47, 3.15)	2.34 (1.69, 3.21)	1.91 (1.39, 3.02)	0.287
Erythrocyte sedimentation rate, mm/h ^a^	12 (6, 22)	15.5 (4.25, 34.75)	12 (6, 21)	0.354
Complement 3, g/L ^b^	0.62 ± 0.19	0.62 ± 0.16	0.62 ± 0.19	0.916
Complement 4, g/L ^b^	0.13 ± 0.06	0.13 ± 0.07	0.13 ± 0.06	0.820

Notes: *p* < 0.05. ^a^: Values are presented as the median (25th and 75th percentiles) and were analyzed by Mann–Whitney U test. ^b^: Values are presented as the mean ± SD and were analyzed by independent *t* test.

**Table 3:** Differences in B-lymphocyte subsets between SLE patients with and without depression.

	SLE Patients	Depression	Non-Depression	*p*
ASC%PBMC	0.26 (0.12, 0.50)	0.71 (0.24, 1.17)	0.24 (0.12, 0.45)	0.002 *
ASC%CD19+	3.88 (1.83, 8.35)	7.42 (4.22, 14.45)	3.43 (1.53, 7.32)	0.011 *
B+%PBMC	5.48 (3.34, 10.01)	6.60 (4.59, 9.45)	5.18 (3.04, 10.65)	0.390
IgA+%ASC	33 (15.88, 46.18)	33.40 (13.83, 55.70)	33 (14.83, 45.95)	0.405
IgAM-%ASC	9.56 (1.52, 18.18)	8.17 (0.57, 15.55)	10.25 (1.65, 18.33)	0.476
IgM+%ASC	39.45 (25.98, 61.85)	35.10 (26.98, 57.03)	42.45 (25.33, 62.55)	0.806
CD138+%ASC	10.8 (6.94, 16.03)	9.84 (6.27, 13.53)	11.85 (7.21, 16.93)	0.332
Bn%B	36.4 (21.23, 49.15)	36.95 (24.08, 51.98)	36.40 (19.88, 47.65)	0.652
Bim%B	7.20 (2.29, 13.20)	11.13 (4.54, 20.40)	6.15 (1.79, 11.75)	0.084
IgM+%B	4.21 (2.33, 6.96)	3.82 (2.91, 5.05)	4.29 (2.24, 7.40)	0.740
IgM+D-%B	0.51 (0.12, 1.08)	0.49 (0.10, 0.73)	0.52 (0.17, 1.16)	0.584
SwBm%B	25.75 (15.48, 43.30)	22.45 (15.55, 41.23)	25.90 (15.23, 46.53)	0.726
IgA+%SwBm	42.95 (19.03, 75.03)	60.05 (22.18, 75.48)	37.95 (18.48, 75.08)	0.631
IgA-%SwBm	49.6 (20.35, 76.25)	30.80 (15.35, 74.18)	54.80 (21.33, 78.43)	0.665
AtM%B	5.10 (2.55, 9.77)	6.34 (3.45, 10.50)	4.81 (2.36, 9.56)	0.607
9G4%Bn	3.67 (2.46, 5.24)	4.41 (3.23, 6.13)	3.55 (2.28, 4.90)	0.149
9G4%Bim	2.72 (1.17, 4.48)	3.69 (1.57, 4.60)	2.69 (1.08, 4.57)	0.284
9G4%IgM+Bm	2.84 (1.95, 4.35)	3.96 (2.39, 4.74)	2.80 (1.73, 4.10)	0.121
9G4%IgM+alone	0 (0, 1.72)	0 (0, 3.48)	0 (0, 1.63)	0.277
9G4%SwBm	2.02 (1.12, 3.37)	2.37 (1.66, 3.46)	1.84 (1.08, 3.33)	0.196
9G4%IgA+	1.73 (0.67, 2.57)	1.92 (0.65, 2.58)	1.68 (0.65, 2.54)	0.784
9G4%IgA-	1.79 (1.03, 2.77)	1.80 (0.86, 2.86)	1.79 (1.04, 2.73)	0.973

Notes: Values are presented as the median (25th and 75th percentiles) and were analyzed by Mann–Whitney U test. *: *p* < 0.05. Abbreviations: PBMC: peripheral blood mononuclear cell; Bn: naïve immature B cells; Btr: transitional B cells; SwBm: switched memory B cell; ASC: plasma B cell; AtM: atypical memory B cells.

**Table 4:** Differences in T-lymphocyte subsets between SLE patients with and without depression.

	SLE Patients	Depression	Non-Depression	*p*
MAIT	0.20 (0.05, 0.75)	0.55 (0.19, 1.62)	0.15 (0.05, 0.62)	0.009 *
TEM%Th	11.65 (3.90, 21.98)	20.15 (11.78, 31.25)	9.15 (3.38, 19.55)	0.004 *
TEMRA%Th	0.37 (0.08, 1.17)	1.02 (0.53, 1.91)	0.26 (0.06, 0.94)	0.003 *
CD45RA+CD27-Th	0.45 (0.20, 1.49)	1.12 (0.43, 2.54)	0.40 (0.19, 1.33)	0.014 *
TEMRA%CD8	17.45 (10.10, 27.03)	22.60 (17.20, 29.38)	14.80 (7.28, 26.35)	0.014 *
γδ1T	1.00 (0.35, 2.19)	1.04 (0.45, 2.02)	0.95 (0.29, 2.20)	0.655
γδ2T	1.10 (0.43, 2.73)	0.82 (0.30, 2.35)	1.11 (0.49, 2.76)	0.420
γδ1-2-T	0.42 (0.21, 0.84)	0.54 (0.39, 1.02)	0.39 (0.20, 0.83)	0.180
iNKT	0.10 (0.04, 0.20)	0.16 (0.10, 0.22)	0.09 (0.03, 0.20)	0.111
αβT	89.95 (85.48, 92.62)	90.10 (85.60, 92.45)	89.85 (85.43, 92.98)	0.939
CD4^+^T	47.10 (39.70, 56.18)	48.00 (43.30, 57.20)	46.00 (39.63, 56.05)	0.445
CD8^+^T	47.65 (37.93, 56.28)	47.80 (37.90, 54.18)	47.45 (37.93, 57.25)	0.697
DPT	0.19 (0.10, 0.38)	0.16 (0.08, 0.26)	0.20 (0.10, 0.40)	0.188
DNT	1.15 (0.61, 1.66)	1.06 (0.73, 1.23)	1.32 (0.59, 1.77)	0.320
Th	96.60 (94.60, 98.00)	96.40 (95.2, 97.50)	96.60 (94.53, 98.15)	0.792
Treg	2.41 (1.26, 3.95)	2.74 (1.63, 3.47)	2.21 (1.22, 4.04)	0.539
Tn%Th	30.60 (20.98, 44.23)	28.05 (19.45, 39.78)	33.80 (21.08, 46.28)	0.286
TCM%Th	51.60 (38.40, 60.80)	50.60 (37.48, 57.78)	51.60 (38.50, 62.38)	0.697
CD27-CD28+Th	10.45 (6.84, 14.85)	11.40 (8.62, 16.55)	10.03 (6.45, 14.28)	0.125
CD27+CD28+Th	81.20 (69.00, 89.05)	78.75 (61.30, 84.63)	82.85 (73.68, 90.20)	0.090
CD27+CD28-Th	0.04 (0.01, 0.13)	0.07 (0.02, 0.15)	0.04 (0.01, 0.13)	0.509
CD27-CD28-Th	5.02 (0.75, 15.25)	7.62 (0.26, 23.63)	5.02 (0.79, 11.78)	0.299
PD1-CD28+Th	71.65 (51.05, 85.53)	64.30 (48.60, 81.48)	76.60 (50.15, 86.20)	0.253
PD1+CD28+Th	14.90 (6.63, 29.50)	15.30 (6.81, 37.33)	14.90 (6.28, 28.68)	0.788
PD1+CD28-Th	1.01 (0.08, 4.32)	1.57 (0.10, 5.74)	0.74 (0.07, 4.29)	0.498
PD1-CD28-Th	1.57 (0.06, 8.92)	2.97 (0.08, 17.93)	1.43 (0.04, 8.13)	0.321
CD45RA-CD27+Th	46.00 (36.03, 52.90)	44.50 (36.25, 54.50)	46.20 (35.88, 52.60)	0.814
CD45RA+CD27+Th	33.90 (20.28, 40.58)	29.75 (19.23, 38.13)	34.50 (20.35, 42.28)	0.334
CD45RA-CD27-Th	17.60 (9.97, 27.60)	23.30 (15.13, 35.68)	16.60 (9.26, 24.88)	0.075
CD45RA-HLADR+Th	20.85 (4.31, 60.95)	12.80 (4.09, 64.23)	23.55 (4.33, 60.48)	0.897
CD45RA+HLADR+Th	1.50 (0.20, 29.98)	0.86 (0.28, 18.75)	4.71 (0.18, 33.30)	0.687
CD45RA+HLADR-Th	10.27 (0.51, 36.78)	19.30 (2.73, 38.85)	7.09 (0.41, 36.48)	0.382
CD45RA-HLADR-Th	31.00 (1.50, 61.55)	48.30 (2.91, 61.55)	18.95 (1.31, 61.73)	0.425
Act%Th	2.84 (0.93, 5.42)	3.64 (2.12, 7.30)	2.59 (0.91, 5.29)	0.205
Tn%Treg	9.37 (3.65, 17.25)	7.34 (3.64, 9.30)	10.55 (3.58, 20.18)	0.071
TCM%Treg	68.30 (51.73, 82.85)	69.40 (52.30, 78.43)	68.30 (51.38, 83.30)	0.855
TEM%Treg	17.40 (7.17, 27.08)	22.05 (16.03, 35.90)	15.30 (5.77, 26.20)	0.055
TEMRA%Treg	0.29 (0.00, 1.06)	0.51 (0.00, 1.47)	0.21 (0.00, 0.96)	0.140
CD27-CD28+Treg	4.91 (2.68, 9.01)	5.22 (2.54, 12.93)	4.49 (2.69, 8.74)	0.676
CD27+CD28+Treg	92.55 (85.20, 96.43)	91.00 (81.85, 97.13)	92.85 (86.63, 96.40)	0.492
CD27+CD28Treg	0.00 (0.00, 0.17)	0.05 (0.00, 0.42)	0.00 (0.00, 0.11)	0.052
CD27-CD28-Treg	0.77 (0.00, 4.48)	1.51 (0.00, 4.79)	0.53 (0.00, 4.16)	0.474
PD1-CD28+Treg	70.70 (48.45, 85.03)	69.15 (47.65, 84.65)	71.40 (48.35, 85.15)	0.817
PD1+CD28+Treg	21.70 (12.08, 48.05)	18.10 (12.08, 47.00)	22.05 (12.03, 50.63)	0.863
PD1+CD28-Treg	0.07 (0.00, 1.79)	0.37 (0.00, 1.79)	0.00 (0.00, 1.86)	0.555
PD1-CD28-Treg	0.00 (0.00, 3.17)	0.67 (0.00, 4.12)	0.00 (0.00, 2.99)	0.146
CD45RA-CD27+Treg	79.80 (63.73, 88.00)	79.60 (69.20, 89.58)	79.85 (59.03, 87.60)	0.498
CD45RA+CD27+Treg	9.13 (3.31, 17.30)	6.24 (4.92, 10.32)	9.77 (2.44, 22.88)	0.178
CD45RA+CD27-Treg	0.00 (0.00, 0.50)	0.00 (0.00, 1.04)	0.00 (0.00, 0.48)	0.652
CD45RA-CD27-Treg	7.62 (3.84, 14.13)	10.14 (4.88, 16.63)	7.46 (3.34, 13.25)	0.206
CD45RA-HLADR+Treg	53.75 (31.25, 81.23)	47.85 (32.90, 76.35)	58.40 (28.95, 82.28)	0.931
CD45RA+HLADR+Treg	1.13 (0.14, 12.33)	1.05 (0.28, 6.33)	1.21 (0.12, 12.78)	0.912
CD45RA+HLADR-Treg	2.23 (0.19, 6.72)	3.89 (1.79, 7.62)	1.87 (0.00, 6.59)	0.264
CD45RA-HLADR-Treg	22.35 (1.83, 58.00)	44.35 (2.79, 58.18)	16.50 (1.50, 57.83)	0.550
Act%Treg	7.67 (3.12, 14.20)	9.81 (4.34, 15.18)	6.68 (2.74, 14.10)	0.507
Act%CD8	2.01 (1.09, 5.49)	4.51 (1.50, 6.92)	1.91 (1.07, 4.68)	0.051
Tn%CD8	39.40 (16.20, 57.93)	31.60 (20.50, 48.55)	40.85 (16.20, 60.63)	0.507
TCM%CD8	4.58 (2.34, 7.05)	3.57 (2.32, 5.23)	4.71 (2.21, 7.86)	0.237
TEM%CD8	31.85 (22.00, 50.58)	35.50 (24.48, 46.73)	30.40 (21.73, 51.18)	0.617
CD27-CD28+CD8	2.43 (1.20, 4.51)	2.52 (1.40, 3.49)	2.43 (1.17, 4.74)	0.878
CD27+CD28+CD8	46.70 (28.80, 65.98)	40.40 (31.10, 58.60)	48.05 (25.10, 66.50)	0.683
CD27+CD28-CD8	5.98 (3.37, 11.05)	8.20 (5.03, 10.93)	5.55 (3.09, 11.20)	0.220
CD27-CD28-CD8	39.35 (24.05, 55.85)	43.45 (31.73, 55.65)	38.30 (23.60, 56.35)	0.474
PD1-CD28+CD8	36.40 (16.28, 60.40)	37.65 (20.90, 55.83)	35.80 (15.23, 62.58)	0.996
PD1+CD28+CD8	4.06 (2.07, 10.75)	3.97 (2.04, 13.33)	4.13 (2.05, 11.05)	0.988
PD1+CD28-CD8	3.71 (1.09, 17.23)	3.57 (1.28, 21.50)	3.71 (0.88, 16.88)	0.504
PD1-CD28-CD8	35.15 (11.48, 57.20)	38.55 (18.33, 55.38)	31.15 (6.86, 58.20)	0.471
CD45RA-CD27+CD8	13.15 (7.50, 18.98)	14.10 (8.14, 18.85)	13.05 (7.44, 19.13)	0.733
CD45RA+CD27+CD8	41.50 (20.70, 56.98)	34.60 (24.88, 50.05)	43.75 (19.58, 60.50)	0.855
CD45RA+CD27-CD8	15.95 (8.13, 24.78)	18.70 (15.30, 25.48)	14.65 (6.92, 24.68)	0.062
CD45RA-CD27-CD8	24.25 (14.88, 37.90)	27.35 (15.50, 32.50)	23.80 (11.60, 38.35)	0.762
CD45RA-HLADR+CD8	5.65 (2.31, 14.45)	7.23 (2.68, 15.60)	5.43 (2.20, 14.00)	0.715
CD45RA+HLADR+CD8	2.23 (0.70, 5.87)	3.28 (0.52, 7.21)	1.96 (0.75, 5.32)	0.669
CD45RA+HLADR-CD8	59.90 (43.08, 70.50)	53.85 (44.83, 67.13)	61.05 (40.83, 71.40)	0.874
CD45RA-HLADR-CD8	28.90 (18.98, 39.55)	28.90 (18.15, 41.03)	29.95 (19.13, 40.23)	0.939

Notes: Values are presented as the median (25th and 75th percentiles) and were analyzed by Mann–Whitney U test. *: *p* < 0.05. Abbreviations: iNKT-cells: invariant natural killer T cells; MAIT: mucosal-associated invariant T cell; DPT: CD4+CD8+ T cells; DNT: CD4-CD8- T cells; Th: helper T cells; Treg: regulatory T cells; Tn: naïve T cells; TCM: central memory T cells; TEM: effective memory T cells; TEMRA: effector memory CD45RA+ T cells; Act: active T cells.

**Table 5:** Logistic analysis of depression in SLE patients.

	B	SE	*p*	Exp (B)	95%CI
Objective support	−0.603	0.144	<0.001 *	0.547	0.412, 0.726
Fatigue	0.049	0.018	0.005 *	1.051	1.015, 1.088
ASC%CD19+	0.067	0.028	0.016 *	1.07	1.013, 1.129

Notes: *: *p* < 0.05, R^2^ = 0.641. Abbreviation: ASC: plasma B cells.

**Table 6:** The weights of different variables in the SVM model.

	Weight		Weight
Sex	0.077	ASC%PBMC	0.085
Fatigue	0.137	ASC%CD19+	0.100
Sleep quality	0.004	MAIT	0.073
Objective support	0.125	TEM%Th	0.170
		TEMRA%Th	0.063
		CD45RA+CD27-Th	0.071
		TEMRA%CD8	0.114

Notes: R^2^ = 0.641. Abbreviations: ASC: plasma B cell; PBMC: peripheral blood mononuclear cell; MAIT: mucosal-associated invariant T cell; TEMRA: effector memory CD45RA+ T cells; Th: helper T cells.

## Data Availability

Data can be obtained by contacting the corresponding author.

## References

[1] Shipa M., Santos L.R., Nguyen D.X., Embleton-Thirsk A., Parvaz M., Heptinstall L.L., Pepper R.J., Isenberg D.A., Gordon C., Ehrenstein M.R. (2023). Identification of biomarkers to stratify response to B-cell-targeted therapies in systemic lupus erythematosus: An exploratory analysis of a randomised controlled trial. Lancet Rheumatol..

[2] Gu X.X., Jin Y., Fu T., Zhang X.M., Li T., Yang Y., Li R., Zhou W., Guo J.X., Zhao R. (2021). Relevant Characteristics Analysis Using Natural Language Processing and Machine Learning Based on Phenotypes and T-Cell Subsets in Systemic Lupus Erythematosus Patients with Anxiety. Front. Psychiatry.

[3] Bingham K.S., DiazMartinez J., Green R., Tartaglia M.C., Ruttan L., Su J., Wither J.E., Kakvan M., Anderson N., Bonilla D. (2021). Longitudinal relationships between cognitive domains and depression and anxiety symptoms in systemic lupus erythematosus. Semin. Arthritis Rheum..

[4] Chalhoub N.E., Luggen M.E. (2022). Depression-, Pain-, and Health-Related Quality of Life in Patients with Systemic Lupus Erythematosus. Int. J. Rheumatol..

[5] Zhao Q., Deng N., Chen S., Cui Y., Du X., Gu Z. (2018). Systemic lupus erythematosus is associated with negatively variable impacts on domains of sleep disturbances: A systematic review and meta-analysis. Psychol. Health Med..

[6] Shen B., Tan W., Feng G., He Y., Liu J., Chen W., Huang X., Da Z., Xu X., Liu H. (2013). The correlations of disease activity, socioeconomic status, quality of life, and depression/anxiety in Chinese patients with systemic lupus erythematosus. Clin. Dev. Immunol..

[7] Wang Y., Zhao R., Gu C., Gu Z., Li L., Li Z., Dong C., Zhu J., Fu T., Gao J. (2019). The impact of systemic lupus erythematosus on health-related quality of life assessed using the SF-36: A systematic review and meta-analysis. Psychol. Health Med..

[8] Shen B., Feng G., Tang W., Huang X., Yan H., He Y., Chen W., Da Z., Liu H., Gu Z. (2014). The quality of life in Chinese patients with systemic lupus erythematosus is associated with disease activity and psychiatric disorders: A path analysis. Clin. Exp. Rheumatol..

[9] Li Z., Yang Y., Dong C., Li L., Cui Y., Zhao Q., Gu Z. (2018). The prevalence of suicidal ideation and suicide attempt in patients with rheumatic diseases: A systematic review and meta-analysis. Psychol. Health Med..

[10] Feng J.Z., Wang Y., Peng J., Sun M.W., Zeng J., Jiang H. (2019). Comparison between logistic regression and machine learning algorithms on survival prediction of traumatic brain injuries. J. Crit. Care.

[11] Barraclough M., Erdman L., Diaz-Martinez J.P., Knight A., Bingham K., Su J., Kakvan M., Munoz Grajales C., Tartaglia M.C., Ruttan L. (2022). Systemic lupus erythematosus phenotypes formed from machine learning with a specific focus on cognitive impairment. Rheumatology.

[12] Bergmans R.S., Loewenstein E., Aboul-Hassan D., Chowdhury T., Schaefer G., Wegryn-Jones R., Xiao L.Z., Yu C., Moore M.N., Kahlenberg J.M. (2023). Social determinants of depression in systemic lupus erythematosus: A systematic scoping review. Lupus.

[13] Chen H., Cui H., Geng Y., Jin T., Shi S., Li Y., Chen X., Shen B. (2022). Development of a nomogram prediction model for depression in patients with systemic lupus erythematosus. Front. Psychol..

[14] Sorensen N.V., Frandsen B.H., Orlovska-Waast S., Buus T.B., Odum N., Christensen R.H., Benros M.E. (2023). Immune cell composition in unipolar depression: A comprehensive systematic review and meta-analysis. Mol. Psychiatry.

[15] Krause D., Stapf T.M., Kirnich V.B., Hennings A., Riemer S., Chrobok A., Fries D.R., Pedrosa Gil F., Rief W., Schwarz M.J. (2018). Stability of Cellular Immune Parameters over 12 Weeks in Patients with Major Depression or Somatoform Disorder and in Healthy Controls. Neuroimmunomodulation.

[16] Miyata S., Yamagata H., Matsuo K., Uchida S., Harada K., Fujihara K., Yanagawa Y., Watanabe Y., Mikuni M., Nakagawa S. (2020). Characterization of the signature of peripheral innate immunity in women with later-life major depressive disorder. Brain Behav. Immun..

[17] Williams E.M., Hyer J.M., Viswanathan R., Faith T.D., Egede L., Oates J.C., Marshall G.D. (2017). Cytokine balance and behavioral intervention; findings from the Peer Approaches to Lupus Self-Management (PALS) project. Hum. Immunol..

[18] Yun Y., Wang X., Xu J., Jin C., Chen J., Wang X., Wang J., Qin L., Yang P. (2023). Pristane induced lupus mice as a model for neuropsychiatric lupus (NPSLE). Behav. Brain Funct..

[19] Fan K.Q., Li Y.Y., Wang H.L., Mao X.T., Guo J.X., Wang F., Huang L.J., Li Y.N., Ma X.Y., Gao Z.J. (2019). Stress-Induced Metabolic Disorder in Peripheral CD4(+) T Cells Leads to Anxiety-like Behavior. Cell.

[20] Andaur Navarro C.L., Damen J.A.A., van Smeden M., Takada T., Nijman S.W.J., Dhiman P., Ma J., Collins G.S., Bajpai R., Riley R.D. (2022). Systematic review identifies the design and methodological conduct of studies on machine learning-based prediction models. J. Clin. Epidemiol..

[21] Zhong M., Zhang H., Yu C., Jiang J., Duan X. (2022). Application of machine learning in predicting the risk of postpartum depression: A systematic review. J. Affect. Disord..

[22] Thibault T., Bourredjem A., Maurier F., Wahl D., Muller G., Aumaitre O., Seve P., Blaison G., Pennaforte J.L., Martin T. (2023). The mediating effect of fatigue in impaired quality of life in systemic lupus erythematosus: Mediation analysis of the French EQUAL cohort. Rheumatology.

[23] Aranow C., Atish-Fregoso Y., Lesser M., Mackay M., Anderson E., Chavan S., Zanos T.P., Datta-Chaudhuri T., Bouton C., Tracey K.J. (2021). Transcutaneous auricular vagus nerve stimulation reduces pain and fatigue in patients with systemic lupus erythematosus: A randomised, double-blind, sham-controlled pilot trial. Ann. Rheum. Dis..

[24] Palagini L., Tani C., Mauri M., Carli L., Vagnani S., Bombardieri S., Gemignani A., Mosca M. (2014). Sleep disorders and systemic lupus erythematosus. Lupus.

[25] Beurel E., Medina-Rodriguez E.M., Jope R.S. (2022). Targeting the Adaptive Immune System in Depression: Focus on T Helper 17 Cells. Pharmacol. Rev..

[26] Pu Y., Zhang Q., Tang Z., Lu C., Wu L., Zhong Y., Chen Y., Hashimoto K., Luo Y., Liu Y. (2022). Fecal microbiota transplantation from patients with rheumatoid arthritis causes depression-like behaviors in mice through abnormal T cells activation. Transl. Psychiatry.

[27] Gascue A., Merino J., Paiva B. (2018). Flow Cytometry. Hematol. Oncol. Clin. North. Am..

[28] Mohd Ashari N.S., Mohamed Sanusi S.N.F., Mohd Yasin M.A., Che Hussin C.M., Wong K.K., Shafei M.N. (2019). Major depressive disorder patients on antidepressant treatments display higher number of regulatory T cells. Malays. J. Pathol..

[29] Varun C.N., Venkataswamy M.M., Ravikumar R., Nagaraju R., Debnath M., Varambally S., Venkatasubramanian G., Ravi V. (2019). Th17 and MAIT cell mediated inflammation in antipsychotic free schizophrenia patients. Schizophr. Res..

[30] Gargano F., Guerrera G., Piras E., Serafini B., Di Paola M., Rizzetto L., Buscarinu M.C., Annibali V., Vuotto C., De Bardi M. (2022). Proinflammatory mucosal-associated invariant CD8+ T cells react to gut flora yeasts and infiltrate multiple sclerosis brain. Front. Immunol..

[31] Cho Y.N., Kee S.J., Kim T.J., Jin H.M., Kim M.J., Jung H.J., Park K.J., Lee S.J., Lee S.S., Kwon Y.S. (2014). Mucosal-associated invariant T cell deficiency in systemic lupus erythematosus. J. Immunol..

[32] Chiba A., Tamura N., Yoshikiyo K., Murayama G., Kitagaichi M., Yamaji K., Takasaki Y., Miyake S. (2017). Activation status of mucosal-associated invariant T cells reflects disease activity and pathology of systemic lupus erythematosus. Arthritis Res. Ther..

[33] Devarajan P., Chen Z. (2013). Autoimmune effector memory T cells: The bad and the good. Immunol. Res..

[34] Sallusto F., Impellizzieri D., Basso C., Laroni A., Uccelli A., Lanzavecchia A., Engelhardt B. (2012). T-cell trafficking in the central nervous system. Immunol. Rev..

[35] Gate D., Saligrama N., Leventhal O., Yang A.C., Unger M.S., Middeldorp J., Chen K., Lehallier B., Channappa D., De Los Santos M.B. (2020). Clonally expanded CD8 T cells patrol the cerebrospinal fluid in Alzheimer’s disease. Nature.

[36] Dubois T., Zdanowicz N., Reynaert C., Jacques D. (2016). Depression, family and immunity: Influence of humoral immunity on family relationships and on the severity of depression. Psychiatr. Danub..

[37] Li Q., Luo Y., Liu D., Li B., Liu Y., Wang T. (2022). Construction and prognostic value of enhanced CT image omics model for noninvasive prediction of HRG in bladder cancer based on logistic regression and support vector machine algorithm. Front. Oncol..

